# Distinct effect of body mass index by sex as a prognostic factor in localized renal cell carcinoma treated with nephrectomy ~ data from a multi-institutional study in Japan ~

**DOI:** 10.1186/s12885-021-07883-9

**Published:** 2021-02-27

**Authors:** Takeshi Tsutsumi, Kazumasa Komura, Takeshi Hashimoto, Ryu Muraoka, Naoya Satake, Tomohisa Matsunaga, Takuya Tsujino, Yuki Yoshikawa, Tomoaki Takai, Koichiro Minami, Kohei Taniguchi, Tomohito Tanaka, Hirofumi Uehara, Hajime Hirano, Hayahito Nomi, Naokazu Ibuki, Kiyoshi Takahara, Teruo Inamoto, Yoshio Ohno, Haruhito Azuma

**Affiliations:** 1 Department of Urology, Osaka Medical College, 2-7 Daigaku-machi, Takatsuki City, Osaka, 569-8686 Japan; 2 Translational Research Program, Osaka Medical College, 2-7 Daigaku-machi, Takatsuki City, Osaka, 569-8686 Japan; 3 Department of Urology, Tokyo Medical University, 6-7-1 Nishi-shinjuku, Shinjuku-ku, Tokyo, 160-0023 Japan; 4 Department of Urology, Fujita-Health University School of Medicine, 1-98 Dengakugakubo, Kutsukake, Toyoake, Aichi Japan

**Keywords:** Renal cell carcinoma, Body mass index, Sex, Prognostic factor

## Abstract

**Background:**

We assessed the prognostic value of body mass index (BMI) in Asian patients with localized RCC who underwent nephrectomy.

**Methods:**

A total of 665 patients who underwent nephrectomy for localized RCC were enrolled in the present study and divided into the two BMI groups: i.e., BMI < 25 in 463 (69.6%) and BMI > 25 in 202 (30.4%) patients.

**Results:**

In total, there were 482 (72.5%) males and 183 (27.5%) females. Five-year cancer-specific survival (CSS) rates were significantly higher in increased BMI than the lower BMI group (97.1 and 92.5%: *P* = 0.007). When stratified by sex, significantly longer CSS in higher BMI was confirmed in males (5-year CSS of 92.7% in BMI < 25 and 98.1% in BMI > 25, *p* = 0.005), while there was no difference in CSS between BMI groups for female patients. Multivariable analysis exhibited that higher BMI was an independent predictor for favorable CSS in male (cox model: *p* = 0.041, Fine & Gray regression model: *p* = 0.014), but not in the female. Subgroup analysis for CSS revealed that favorable CSS with higher BMI was observed in patient subgroups of age < 65 (*p* = 0.019), clear cell histology (*p* = 0.018), and tumor size > 4 cm, *p* = 0.020) as well as male (*p* = 0.020).

**Conclusion:**

Our findings collected from the multi-institutional Japanese dataset demonstrated longer survival in patients with higher BMI than lower BMI for non-metastatic RCC treated with nephrectomy. Intriguingly, this finding was restricted to males, but not to females.

## Background

Renal cell carcinoma (RCC) is the most common kidney cancer, and expected numbers in the United States account for 65,340 new cases and 14,970 deaths in 2018 [1]. A number of risk factors of developing RCC have been reported, including smoking, hypertension, sex, and obesity [2]. Although obesity is a well-known factor in developing RCC, several studies have indicated that obese patients treated with surgery for RCC may have a more favorable prognosis [3–7]. Recently, Albiges et al. further demonstrated that a multicenter cohort involving 1975 patients from the International Metastatic Renal Cell Carcinoma Database Consortium (IMDC) and an external validation cohort of 4657 patients revealed an improved survival in patients with higher body mass index (BMI) treated with molecular targeted agents for metastatic RCC [8]. However, whether these findings from the Caucasian population consistently can be applied to all races/ethnicity is still unknown. For example, a recent study suggested that RCC in Hispanic Americans and Native Americans have different clinical characteristics compared with European American patients [9, 10]. With regard to the Asian patients, the incidence of RCC seems to be less frequent in the Asian population than Caucasian, and treatment outcomes may differ between these ethnicities suggesting that the role of prognostic factors including BMI varies between ethnicities [11, 12]. In addition, several recent studies indicated that sex might affect the prognostic value of BMI in RCC [13, 14]. We previously reported the value of BMI as a prognostic factor in RCC treated with nephrectomy in the Asian patient cohort [15]. In the present study, we further assess the prognostic value of BMI using the multicenter-cohort dataset for the clinically localized RCC in Japanese patients who underwent nephrectomy with curative intent.

## Methods

Between 1987 and 2017, 760 RCC patients underwent either radical or partial nephrectomy in our multicenter cohort, of which clinicopathological data in 665 localized RCC patients with pT1–4 tumors without nodal and distant metastases at surgery were collected. Data were collected from two leading hospitals, i.e., Tokyo Medical University (349 patients: located in Shinjuku-ku, Tokyo) and Osaka Medical College with two affiliated hospitals (316 patients: Osaka Medical College located in Takatsuki city, Saiseikai-Nakatsu Private Hospital located in Osaka city, and Hirakata Municipal Hospital located in Hirakata city, Osaka). Patients who did not undergo nephrectomy or had any missing clinicopathological/laboratory information were excluded from the study. The study design was approved by the institutional review board (IRB approval number: RIN-750-2571) and performed in accordance with the ethical standards of the World Medical Association Declaration of Helsinki [16].

The clinical-stage in each patient was evaluated by computed tomography (CT), magnetic resonance imaging (MRI), ultrasound, and chest-X ray, and other patient information including performance status (Eastern Cooperative Oncology Group, ECOG-PS), BMI was preoperatively recorded within 1 month before surgery. BMI was calculated as the patient’s weight at admission (in kilograms) divided by the patient’s height squared (in meters) and categorized based on WHO recommendations for Asians [17]. Pathological review, including Fuhrman nuclear grade [18] was examined in all patients as well as the 7th TNM classification of the UICC and AJCC guidelines of renal tumors. After discharge, follow-up CT and Chest X-rays were performed to detect any findings suspected of disease progression every 3 months in the first year. Thereafter, patients were followed up every 6 months. Overall survival (OS) and cancer-specific survival (CSS) after nephrectomy were evaluated in all 665 patients. Cancer-specific mortality was defined as death from RCC, not including other cancers. The record of the event was captured from the patient summary at each institute. Follow-up was calculated from the day of surgery to the day of death or the last visit. Recurrence-free survival (RFS) was calculated from the date of surgery to the date of disease recurrence or metastasis or the last follow-up in localized RCC patients.

The distribution of each factor was assessed by a contingency table with a Chi-square analysis. Kolmogorov-Smirnov normality was examined to check normal distribution in continuous variables followed by conducting a student’s t-test, or one-way ANOVA was examined to assess the difference between the variables. For variables with non-normal distribution, Wilcoxon or Kruskal-Wallis test was performed to assess the difference. A Kaplan-Meier analysis was carried out to estimate the survival free ratio, and a log-rank test was performed to compare the difference between assigned patient groups. On multivariable analysis, Cox proportional-hazard regression models and Fine & Gray regression model [19] were utilized. In the Fine & Gray regression model, the strength of the prognostic correlation between variables and cancer-specific mortality was assessed using the sub-hazard ratio that is the hazard ratio associated with the cumulative incidence function (CIF). In all statistical analyses, a 2-sided *p*-value of < 0.05 was considered significant. All analyses were performed using JMP® 13 (SAS Institute Inc., Cary, NC, USA) and the R 4.0.2 software package.

## Results

Table 1 summarizes the clinical and pathologic characteristics of 665 patients according to BMI subgroups (< 25 kg/m2 and > 25 kg/m2). There were 482 (72.5%) and 183 (27.5%) in male and female, respectively. Mean age in all patients was 62.2 ± 12.0 years (range: 21–91). The median follow-up time was 78.0 and 52 months for patients who survived (*n* = 561) and deceased (*n* = 104) during follow-up, respectively. Of the patients who deceased during follow-up, 62 (9.3%) patients died of RCC, 42 (6.3%) had died of other causes. During follow-up, 126 (18.9%) patients developed disease recurrence. The ECOG performance status was 0 in 612 patients (92.0%), 1 in 37 (5.6%), 2 in 13 (2.0%) and > 3 in 3 (0.4%). The histologic subtype of RCC was clear cell in 560 patients (84.2%), papillary in 73 (11.0%), chromophobe in 11 (1.7%) and others in 21 (3.1%). Pathological stage included pT1 in 520 patients (78.2%), pT2 in 61 (9.2%), pT3 in 80 (12.0%), pT4 in 4 (0.6%). Median tumor size was 4 cm (range: 0.9–18). The mean BMI (± SD) was 23.6 ± 3.2 kg/m2 (range: 13–39.8) in the total cohort. There were 463 (69.6%) and 202 (30.4%) patients with BMI of < 25 kg/m2 and > 25 kg/m2, respectively. No significant difference in the distribution of patient characteristics was seen between BMI groups in sex, histological subtypes, pathological stage and tumor size, whereas but there was a significant difference in age (< 65 vs ≥65, *p* = 0.015) and ECOG-PS (0 vs > 1, *p* = 0.012) between BMI groups.

**Table 1:** Clinicopathological characteristics in 655 patients with localyzed RCC

Variables	Total (%)	BMI		*P* value
		<25	≥25	
No. of patients	665	463 (69.6)	202 (30.4)	
Sex				
Male	482 (72.5)	332 (71.7)	150 (74.3)	0.498
Female	183 (27.5)	131(28.3)	52 (25.7)	
Age				
< 65	354 (53.2)	232 (50.1)	122(60.4)	0.015
≥ 65	311 (46.8)	231(49.9)	80 (39.6)	
ECOG-PS				
0	612 (92.0)	418 (90.3)	194 (96.0)	0.012
≥ 1	53 (8.0)	45 (9.7)	8 (4.0)	
Histological type				
clear cell	560 (84.2)	380 (82.1)	180 (89.1)	0.116
papillary	57 (11.0)	57 (2.3)	16 7.9)	
chromophobe	11 (1.7)	8 (1.7)	3 (1.5)	
others	21 (3.2)	18 (3.9)	3 (1.5)	
Pathological stage				
1	520 (78.2)	357 (77.1)	163 (80.7)	0.378
2	61 (9.2)	42 (9.1)	19 (9.4)	
3	80 (12.0)	60 (13.0)	20 (9.9)	
4	4	4 (0.9)	0 (0)	
Tumor size				
< 4cm	366 (55.1)	252 (54.4)	114 (56.7)	0.586
≥ 4cm	298 (44.9)	211 (45.6)	87 (43.3)	

*RCC* renal cell carcinoma, *BMI* body mass index, *ECOG-PS* eastern cooperative oncology group - performance status

**Fig. 1:**
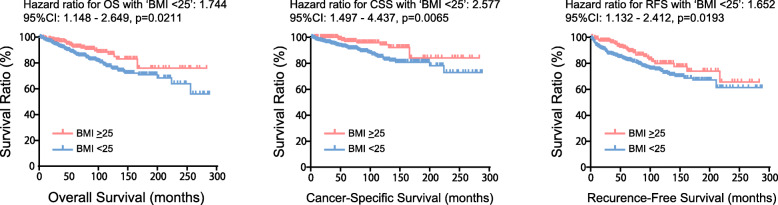
Kaplan-Meier curves of OS, CSS, and RFS in 655 localized RCC patients according to BMI subgroups

**Fig. 2:**
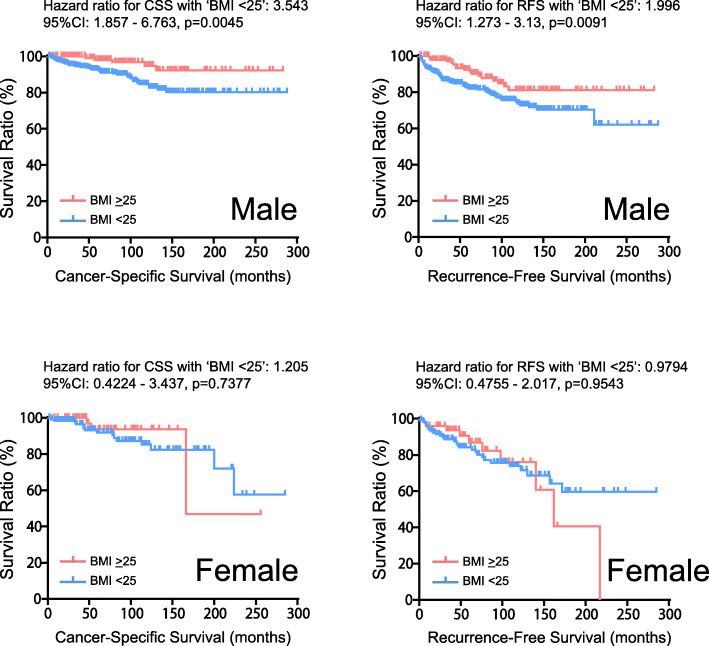
Kaplan-Meier curves of CSS and RFS in localized RCC patients according to BMI subgroups with the stratification by sex

Kaplan-Meier curves showed significantly longer OS in patients with higher BMI, in which the 5-year OS rates in BMI < 25 kg/m2 and ≥ 25 kg/m2 groups were 87.3 and 92.6%, respectively (*P* = 0.021) (Fig. 1). We also assessed CSS. As expected, the 5-year CSS and RFS rate was more favorable in higher BMI (97.1 and 91.1%) compared to lower BMI group (92.5 and 82.7%) (*P* = 0.007 for CSS, and *p* = 0.019 for RFS), suggesting the prognostic value of BMI in patients with RCC treated with nephrectomy. Of note, when stratified by sex as shown in Fig. 2, significantly longer CSS in higher BMI was confirmed in male (5-year CSS of 92.7% in BMI < 25 and 98.1% in BMI > 25, *p* = 0.005), while there was no difference in CSS between BMI groups for female patients (5-year CSS of 91.9% in BMI < 25 and 93.7% in BMI > 25, *p* = 0.738). Longer RFS in higher BMI group was also observed in male patients (5-year RFS of 82.1% in BMI < 25 and 92.4% in BMI > 25, *p* = 0.009), but not in female (5-year RFS of 84.2% in BMI < 25 and 86.9% in BMI > 25, *p* = 0.954).

**Fig. 3:**
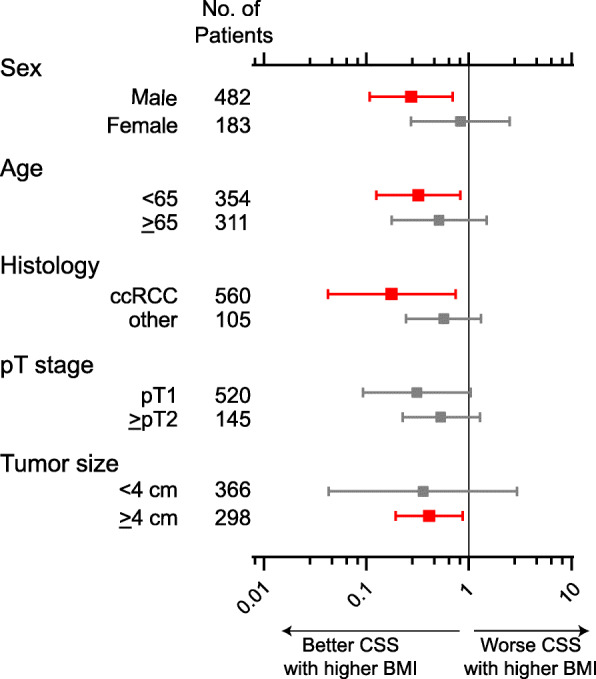
The forest plots of subgroup analysis of CSS according to BMI in 665 localized RCC patients

To further interrogate the prognostic value of putative variables affecting CSS including BMI, we conducted multivariable analyses using the Cox regression model as well as the Fine & Gray regression model that offers sub-hazard ratio (SHR) by weighing the competing risk of death with other cause (Table 2). Increased BMI was as an independent prognostic factor of longer CSS in both cox regression (HR: 0.48, 95%CI: 0.24–0.98, *p* = 0.045) and Fine & Gray regression model (SHR: 0.3, 95%CI: 0.11–0.85, *p* = 0.023). Next, to assess whether the prognostic value of BMI is associated with sex, we separately examined the regression model analyses to predict CSS according to sex (Table 3). Multivariable analysis revealed that BMI still remains as an independent predictor for CSS in male (cox model; HR: 0.37, 95%CI: 0.14–0.96, *p* = 0.041, Fine & Gray model; HR: 0.2, 95%CI: 0.06–0.72, *p* = 0.014), but not in female (cox model; *p* = 0.65, Fine & Gray model; *p* = 0.518). Finally, we conducted subgroup analysis for cancer-specific mortality according to BMI in 665 localized RCC patients (Fig. 3), which revealed that favorable CSS with higher BMI was observed in patient subgroups of age < 65 (HR: 0.32, 95%CI: 0.13–0.83, *p* = 0.019), ccRCC (HR: 0.12, 95%CI: 0.04–0.75, *p* = 0.018), and tumor size > 4 cm (HR: 0.41, 95%CI: 0.19–0.87, *p* = 0.020) as well as male (HR: 0.41, 95%CI: 0.19–0.87, *p* = 0.020). Since the majority of RCC was diagnosed with clear cell RCC (ccRCC: 560/665 patients) that is found to have a worse prognosis compared to other histological subtypes [20], we performed a multivariable analysis in 560 ccRCC patients (Table 4). Increased BMI seemed to be an independent predictor for favorable CSS in male (cox model; HR: 0.39, 95%CI: 0.15–1.04, *p* = 0.059, Fine & Gray model; HR: 0.22, 95%CI: 0.06–0.80, *p* = 0.022), but not in female (cox model; *p* = 0.398, Fine & Gray model; *p* = 0.527).

**Table 2:** Multivariable analysis using cox regression and Fine&Gray regression models for predicting CSS in 655 patients with localyzed RCC

Variables	Cox regression model			Fine&Gray regression model		
	OR	95% CI	*P* value	SHR	95% CI	*P* value
Age (<65 vs ≥65 years)	1.05	0.62-1.79	0.848	0.73	0.39-1.36	0.32
ECOG Performance Status (0 vs ≥1)	4.08	2.18-7.62	<0.001*	4.02	2.06-7.82	<0.001*
Histological type (Clear cell vs others)	1.15	0.50-2.60	0.746	0.74	0.28-1.95	0.546
Pathological stage (1-2 vs >3)	3.94	2.30-6.74	<0.001*	4.15	2.22-7.74	<0.001*
Tumor size (<4 vs ≥4cm)	6.00	2.66-13.52	<0.001*	4.41	1.77-11.02	0.001*
BMI (<25 vs >25)	0.48	0.24-0.98	0.045*	0.3	0.11-0.85	0.023*

*CSS* cancer-specific survival, *OR* odds rario, *CI* confidence interval, *SHR* sub-hazard ratio, *ECOG* eastern cooperative oncology group, *BMI* body mass index, *denotes *p* < 0.05

**Table 3:** Multivariable analysis stratifying by sex using cox regression and Fine&Gray regression models for predicting CSS in 665 localized RCC patients

Variables	Male						Female					
	Cox regression model			Fine&Gray regression model			Cox regression model			Fine&Gray regression model		
	HR	95% CI	*P* value	SHR	95% CI	*P* value	HR	95% CI	*P* value	SHR	95% CI	*P* value
Age (<65 vs ≥65 years)	0.74	0.38-1.45	0.381	0.55	0.25-1.19	0.127	2.20	0.80-6.01	0.124	0.71	0.15-3.36	0.665
ECOG Performance Status (0 vs ≥1)	4.28	1.86-9.86	<0.001*	4.13	1.74-9.76	0.001*	5.77	1.91-17.46	0.002*	4.21	1.33-13.36	0.015*
Histological type (Clear cell vs others)	0.79	0.27-2.35	0.67	0.80	0.24-2.66	0.717	3.19	0.84-12.15	0.089	0.70	0.12-3.90	0.68
Pathological stage (1-2 vs >3)	4.20	2.18-8.08	<0.001*	4.34	2.04-9.24	<0.001*	3.20	1.17-8.75	0.024*	3.93	1.10-13.96	0.035*
Tumor size (<4 vs ≥4cm)	8.98	3.13-25.75	<0.001*	4.14	1.57-10.95	0.004*	2.28	0.58-9.02	0.238	10.44	0.91-119.2	0.059
BMI (<25 vs >25)	0.37	0.14-0.96	0.041*	0.20	0.06-0.72	0.014*	0.76	0.23-2.48	0.65	0.52	0.07-3.77	0.518

*CSS* cancer-specific survival, *OR* odds rario, *CI* confidence interval, *SHR* sub-hazard ratio, *ECOG* eastern cooperative oncology group, *BMI* body mass index, * denotes *p* < 0.05

**Table 4:** Multivariable analysis stratifying by sex using cox regression and Fine&Gray regression models for predicting CSS in 560 localized RCC patients diagnosed with clear cell carcinoma

Variables	Male						Female					
	Cox regression model			Fine&Gray regression model			Cox regression model			Fine&Gray regression model		
	HR	95% CI	*P* value	SHR	95% CI	*P* value	HR	95% CI	*P* value	SHR	95% CI	*P* value
Age (<65 vs ≥65 years)	0.74	0.36-1.49	0.396	0.53	0.23-1.21	0.134	1.69	0.59-4.81	0.324	0.45	0.06-3.36	0.438
ECOG Performance Status (0 vs ≥1)	4.59	1.84-11.45	0.001	4.49	1.81-11.13	0.001*	5.97	1.77-20.17	0.004*	4.60	1.09-19.40	0.038*
Pathological stage (1-2 vs >3)	4.26	2.13-8.50	<0.001*	4.45	2.02-9.81	<0.001*	3.17	1.07-9.37	0.037*	5.60	1.03-30.32	0.046*
Tumor size (<4 vs ≥4cm)	7.62	2.64-21.94	<0.001*	3.62	1.39-9.37	0.008*	2.77	0.57-13.56	0.208	22.86	1.05-499.7	0.047*
BMI (<25 vs >25)	0.39	0.15-1.04	0.059	0.22	0.06-0.80	0.022*	0.57	0.15-2.12	0.398	0.47	0.04-4.93	0.527

*CSS* cancer-specific survival, *OR* odds rario, *CI* confidence interval, *SHR* sub-hazard ratio, *ECOG* eastern cooperative oncology group, *BMI* body mass index, *denotes *p* < 0.05

## Discussion

Obesity has been recognized as a risk factor for various diseases. To date, a number of epidemiological and clinical studies have suggested that obesity is a significant risk factor for developing RCC. Renehan et al. reported a systematic review of 221 databases to uncover the association between obesity and the occurrence of cancer [2]. They demonstrated that a 5 kg/m2 increase in BMI was strongly associated with the risk of RCC in both men (HR: 1.24, *p* < 0.0001) and women (HR: 1.34, *p* < 0.0001). Intriguingly, there have also been several studies that showed a favorable clinical outcome in RCC patients with increased BMI compared to decreased BMI, which is known as the “obesity paradox”, namely higher incidence and improved clinical outcome of RCC in higher BMI population [3, 6, 21]. In 1991, Yu et al. firstly investigated the prognosis of 360 RCC patients at 29 hospitals in Oklahoma between 1981 and 1987, and the disease-free survival and OS were significantly longer in patients who were obese than in non-obese patients [21]. Thereafter, the finding of improved clinical outcome in higher BMI patients for RCC have been supported in considerable data from retrospective studies. In 2016, Donin and colleagues showed the data from a prospective randomized trial reporting an association between obesity and improved overall survival for clear cell RCC [22]. These data were further supported in metastatic RCC in the recent large cohort study, which concludes that higher BMI is a prognostic factor for improved survival and progression-free survival in patients with metastatic RCC treated with targeted therapy [8]. However, these findings were mainly derived from the Caucasian population, which raises the question that BMI can also be applied to all races/ethnicity. For example, the report from Donin et al. stratified BMI into < 25, 25–29.9, 30.0–34.9, and ≥ 35 [22]. Compared with patients with BMI < 30, patients with a BMI ≥ 30 had significantly improved OS in their prospective study. Of note, If we stratified our cohort according to BMI referring to their definition, there were 463 (69.6%), 169 (25.4%), 31 (4.7%), and 2 (0.3%) patients with BMI < 25, 25–29.9, 30.0–34.9, and > 35 (namely, 95% patients assigned to BMI < 30). This is in line with the report from Matsuzawa et al., in which BMI ≥30 is approximately 2–3% in the Japanese population, in contrast to 10–20% in Europe and the United States [23]. Another Japanese study by Hozawa et al. assessed the association between BMI and all-cause death in Japan using thirteen epidemiology-cohorts, and they reported that all-cause mortality risk was lowest in BMI of 22.0–24.9 [24]. In fact, the Yoden index for the optimal cutoff to best predict cancer-specific mortality in our cohort of 665 localized RCC patients was 23.9 of BMI that offers 0.7 in sensitivity and 0.54 in 1-specificity. Therefore, given the different BMI distribution in Japanese from Caucasians, we defined a cutoff point of 25 for BMI in the present study. In the Asian population, reports from Korean cohort studies consistently demonstrated improved clinical outcomes in higher BMI patients [6, 25, 26]. In Japanese RCC patients, several articles interrogating the prognostic value of BMI have been reported, all of which were conducted as a single-institute cohort study [14, 15, 27]. In the current study, we conducted a multi-institutional cohort study for localized RCC patients treated with radical or partial nephrectomy. Consistent with previous studies, increased BMI was significantly correlated with improved clinical outcomes compared to decreased BMI and remained an independent predictor for longer CSS in patients with non-metastatic RCC treated with nephrectomy. Our data also support the hypothesis that the prognostic value of BMI is male-specific, as suggested by Byun et al. [13]. In their study, male patients had a higher BMI ratio than female patients (*P* = 0.03), whereas, in the present study, there was no significant difference in the distribution between BMI groups and sex, which allowed us to assess the crude effect of BMI on prognosis according to sex difference. Furthermore, our subgroup analysis for CSS according to BMI (Fig. 3) suggests that favorable CSS with higher BMI is more likely to be observed in patient subgroups of age < 65, ccRCC, and tumor size > 4 cm as well as being male.

Although several studies have sought to elucidate the biological underpinnings, a mechanism by which obesity may improve clinical outcomes in RCC remains unclear. Adipose tissue produces a variety of inflammatory factors, including leptin, adiponectin, and cytokines. Of them, leptin has been shown to upregulate the expression of phosphorylated-STAT3 (signal transducers and activators of transcription 3), phosphorylated-ERK (extracellular signal-regulated kinase), and AP-1 (transcript activator protein 1), which might confer the proliferative effect on tumor cells [28]. On the other hand, there was a conflicting study showing that serum leptin level was positively correlated with BMI and inversely related to tumor stage and grade [29]. Given the multiple roles of leptin in chronic inflammation and autoimmunity [30], further experiments are required to answer the question. Ito and colleagues recently assessed the impact of BMI, serum adiponectin level, total adiponectin secretion from perinephric adipose tissue, and intratumor expression of adiponectin receptors in RCC [31]. In their study, secreted adiponectin levels in perinephric adipose tissue and intratumor adiponectin receptors (AdipoR1/R2) expression were not correlated with RCC aggressiveness or survival, whereas decreased BMI and increased serum adiponectin level was significantly associated with poor overall survival in patients with non-metastatic RCC, which might offer new molecular insight of ‘obese paradox’. Finally, The Cancer Genome Atlas (TCGA) data set revealed the downregulation of fatty acid synthase (FASN) in obese RCC patients by transcriptome analysis without specific DNA alternation [32]. They demonstrated that increased FASN mRNA expression level was associated with lower BMI and shorter OS. Furthermore, in the IMDC biospecimen cohort, FASN immunohistochemistry positivity was significantly more detected in IMDC poor (48%) and intermediate (34%) risk groups than in the favorable risk group (17%), indicating the potential role of FASN regulating lipid homeostasis in RCC [8].

The present study had some limitations. Firstly, the patient selection was biased as the cohort in the study was retrospectively designed. Secondly, we could not assess potential prognostic factors, such as smoking, molecular markers, and peripheral blood measurement at surgery [33–35]. In particular, smoking is a well-accepted risk factor for RCC development regardless of sex [36]. In addition, the prognosis for RCC patients with current or former smoking history appears to be poorer than never smokers [37] [38]. Thus, it is plausible that smoking status is a significant confounder when assessing the prognostic value of BMI stratifying with males and females. Unfortunately, our cohort dataset does not have a record of smoking status. Nevertheless, our findings collected from multi-institutional Japanese data sets further confirmed the improved survival in patients with higher BMI compared to lower BMI for non-metastatic RCC treated with nephrectomy, and intriguingly, this finding was restricted to male, but not to female. Furthermore, given that other subgroups such as younger (age < 65) and/or tumor size > 4 cm are privileged to have a favorable effect on clinical survival with higher BMI, these findings potentially help physicians for decision making such as operation approach (total or partial nephrectomy). Further research is warranted to unveil the biological mechanisms responsible for the benefit of high BMI on improved RCC survival in males.

## Conclusion

Our findings collected from the multi-institutional Japanese dataset demonstrated longer survival in patients with higher BMI than lower BMI for non-metastatic RCC treated with nephrectomy. Intriguingly, this finding was restricted to males, but not to females.

## Data Availability

The datasets generated during and/or analyzed during the current study are available from the corresponding author on reasonable request.

## Abbreviations

BMI: Body mass index
RCC: Renal Cell Carcinoma
CSS: Cancer-specific survival
IMDC: International Metastatic Renal Cell Carcinoma Database Consortium
CT: Computed tomography
MRI: Magnetic resonance imaging
ECOG-PS: Eastern Cooperative Oncology Group performance status
WHO: World Health Organization
OS: Overall survival
RFS: Recurrence-free survival
CIF: Cumulative incidence function
SHR: Sub-hazard ratio
STAT3: Signal transducers and activators of transcription 3
ERK: Extracellular signal-regulated kinase
AP-1: Transcript activator protein 1
AdipoR: Adiponectin receptors
TCGA: The Cancer Genome Atlas
FASN: Fatty acid synthase
